# Provincial and Territorial Variation in Barriers in Accessing Healthcare for Children and Youth With Mental and Neurodevelopmental Health Concerns in Canada

**DOI:** 10.1177/07067437221114005

**Published:** 2022-08-07

**Authors:** Jordan Edwards, Mahdis Kamali, Stelios Georgiades, Charlotte Waddell, Katholiki Georgiades

**Affiliations:** 1 Department of Psychiatry and Behavioural Neurosciences, 3710 McMaster University, Hamilton, Ontario, Canada; 2 Offord Centre for Child Studies, 3710 McMaster University, Hamilton, Ontario, Canada; 3 Children’s Health Policy Centre, Faculty of Health Sciences, Simon Fraser University, Vancouver, British Columbia, Canada

**Keywords:** child and adolescent psychiatry, access to care, barriers to treatment, provincial and territorial variation

## Introduction

Mental and neurodevelopmental disorders are the two leading contributors to morbidity worldwide.^1,2^ Both classes of disorders emerge early in the life course and are associated with delays in social, and emotional development, burdening those affected and society as a whole.^2–4^ Early intervention is critical for preventing worsening symptoms and impairment and achieving optimal outcomes.^3^ Evidence suggests that many Canadian children and youth (aged 1–17), particularly those with mental health and developmental difficulties, experience barriers to accessing healthcare, which are disproportionately distributed across population subgroups and by class of disorder.^3,5^ In particular, females, immigrants and refugees, Indigenous and racialized subgroups, low-income households, rural residents, and members of the LGBTQ + community may experience greater barriers to accessing care for mental and neurodevelopmental concerns.^5–7^

A strategic priority of the Mental Health Commission of Canada is establishing equity in mental health care delivery for children and youth.^6^ Indicators used to evaluate equitable service delivery include measures of self-reported barriers to accessing care, which incorporates aspects of both need and service delivery. As mental health care is provincially/territorially mandated in Canada, there is also variation in service delivery, which in turn may lead to variations in how children and youth are able to access care. Prior evidence on barriers to care in Canada is sparse.^5^ Yet, recent evidence from the 2019 Canadian Health Survey on Children Youth (CHSCY) provides an opportunity to address data gaps by quantifying the prevalence of barriers in accessing care for mental and neurodevelopmental health concerns among children and youth across all provinces and territories and examining differences in prevalence according to population characteristics.^8^

## Methodology

*Sample:* The 2019 CHSCY is a cross-sectional national survey designed to collect information on issues affecting the physical, mental, and developmental health of children and youth.^8^ The CHSCY provides a nationally representative sample of 47,871 young people aged 1–17 years as of January 31, 2019 and living in the 10 provinces and the territories (3 territories were combined for Statistics Canada’s data disclosure regulations). Sampling is based on the 2018 Canadian Child Benefit file, which provides coverage for 98% of children and youth living in the provinces and 96% in the territories.

*Measurement:* The 2019 CHSCY contains parental reporting for the full age range.^8^ Parents were asked if their child required or received services in the past 12 months for a mental health concern by endorsing yes for either (“mental health issues”, “difficulties focusing or controlling behaviour”) or a neurodevelopmental health concern by endorsing yes for either (“speech or language difficulties”, “learning difficulties”). If parents endorsed yes, they were asked if they experienced any or multiple barriers (“wait times too long”, “service not available in area”, “cost”, “told child not eligible”, and “other reason”) to care for each category. Clinical and sociodemographic factors were also assessed (online Supplemental material).

*Analysis:* The prevalence of barriers was modeled using modified Poisson regression analyses.^9^ We contrasted predictive margins to compare individual provinces/territory to the Canadian average. All models included standardized survey weights and were adjusted for sociodemographic and clinical factors.

## Findings

The prevalence of children and youth requiring or receiving services for mental and neurodevelopmental health-related concerns was 9.91% (95%CI, 9.64 to 10.17) and 11.65% (95%CI, 11.36 to 11.94), respectively. Our findings suggest that approximately 35.8% (mental health concerns) and 31.7% (neurodevelopmental health concerns) of children and youth requiring or receiving services reported barriers to accessing care.

**Figure 1.**
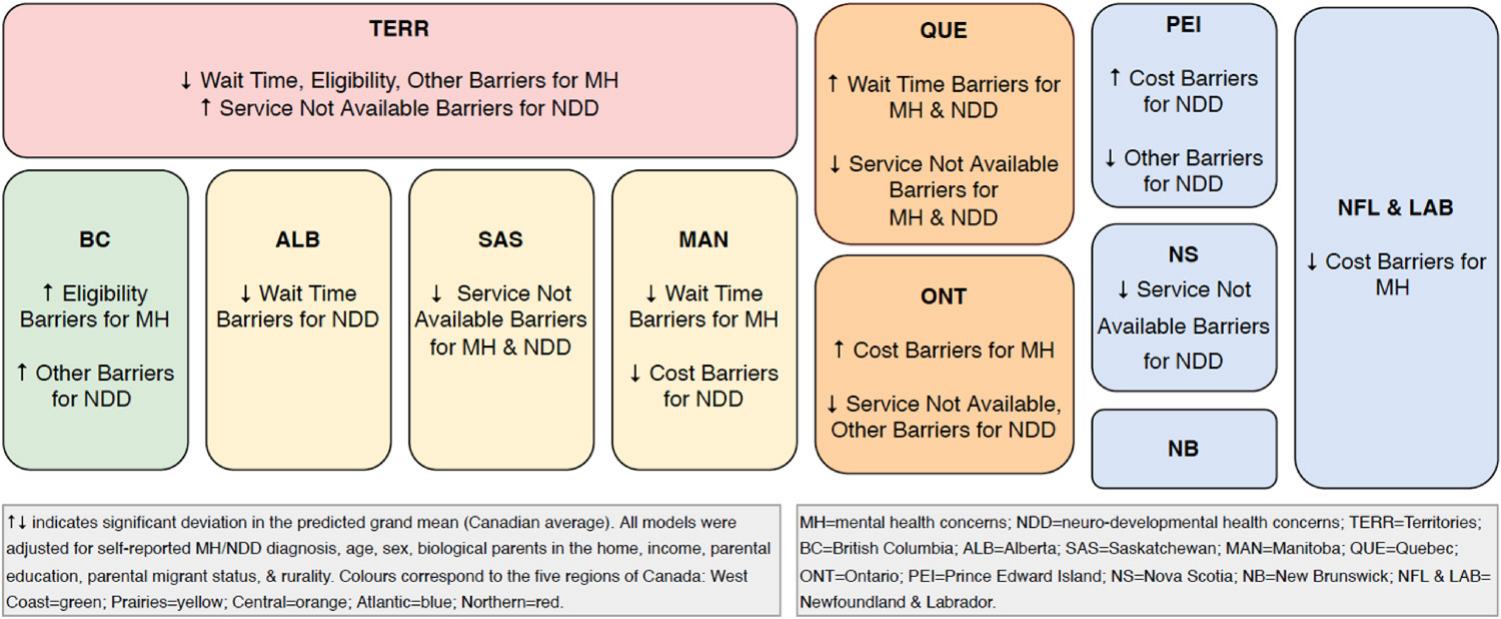
Provincial/territorial variation in barriers to accessing health care for children and youth with mental and neurodevelopmental conditions.

Compared to the national average, we found no provincial variation in barriers to accessing care for both mental and neurodevelopmental health concerns, when barriers were considered as a global composite. However, we found significant variation when analyzing specific types of barriers. Compared to the national average, barriers to accessing care for mental health concerns were more frequent when related to wait times in Quebec, eligibility in British Columbia, and cost in Ontario. Furthermore, we identified that barriers to accessing care for neurodevelopmental health concerns were more frequent when related to wait times in Quebec, cost in Prince Edward Island, service availability in the Territories, and other reasons in British Columbia. Many of these increased barriers were offset with reduced barriers in other characteristics of service access (Figure 1). Findings from our analyses can be found in online Supplemental material.

Our findings are not representative of all Canadian populations with specific data gaps existing for children and youth living on First Nation reserves and in other Aboriginal settlements, those living in foster homes or who are homeless, and institutionalized populations—all of whom may differentially experience barriers to accessing care. We, therefore, believe there is a need to fill this critical knowledge gap in future research.

## Conclusion

This work highlights the use of Canada-wide survey data for making efficient national comparisons of barriers to accessing care for mental and neurodevelopmental health concerns among children and youth. Our findings can serve to support evidence-informed policy and practice and can facilitate evaluations across Canada to monitor progress in reducing inequities in accessing healthcare for mental and neurodevelopmental health concerns among Canadian children and youth. To better understand the policy implications of our findings, we believe future work pairing all available evidence on barriers to accessing care across Canada with an in-depth policy mapping and service delivery analysis is warranted.

## Supplemental Material

sj-docx-1-cpa-10.1177_07067437221114005 - Supplemental material for Provincial and Territorial Variation in Barriers in Accessing Healthcare for Children and Youth With Mental and Neurodevelopmental Health Concerns in Canada Click here for additional data file. Supplemental material, sj-docx-1-cpa-10.1177_07067437221114005 for Provincial and Territorial Variation in Barriers in Accessing Healthcare for Children and Youth With Mental and Neurodevelopmental Health Concerns in Canada by Jordan Edwards, Mahdis Kamali, Stelios Georgiades, Charlotte Waddell and Katholiki Georgiades in The Canadian Journal of Psychiatry

sj-docx-2-cpa-10.1177_07067437221114005 - Supplemental material for Provincial and Territorial Variation in Barriers in Accessing Healthcare for Children and Youth With Mental and Neurodevelopmental Health Concerns in Canada Click here for additional data file. Supplemental material, sj-docx-2-cpa-10.1177_07067437221114005 for Provincial and Territorial Variation in Barriers in Accessing Healthcare for Children and Youth With Mental and Neurodevelopmental Health Concerns in Canada by Jordan Edwards, Mahdis Kamali, Stelios Georgiades, Charlotte Waddell and Katholiki Georgiades in The Canadian Journal of Psychiatry

sj-docx-3-cpa-10.1177_07067437221114005 - Supplemental material for Provincial and Territorial Variation in Barriers in Accessing Healthcare for Children and Youth With Mental and Neurodevelopmental Health Concerns in Canada Click here for additional data file. Supplemental material, sj-docx-3-cpa-10.1177_07067437221114005 for Provincial and Territorial Variation in Barriers in Accessing Healthcare for Children and Youth With Mental and Neurodevelopmental Health Concerns in Canada by Jordan Edwards, Mahdis Kamali, Stelios Georgiades, Charlotte Waddell and Katholiki Georgiades in The Canadian Journal of Psychiatry

sj-docx-4-cpa-10.1177_07067437221114005 - Supplemental material for Provincial and Territorial Variation in Barriers in Accessing Healthcare for Children and Youth With Mental and Neurodevelopmental Health Concerns in Canada Click here for additional data file. Supplemental material, sj-docx-4-cpa-10.1177_07067437221114005 for Provincial and Territorial Variation in Barriers in Accessing Healthcare for Children and Youth With Mental and Neurodevelopmental Health Concerns in Canada by Jordan Edwards, Mahdis Kamali, Stelios Georgiades, Charlotte Waddell and Katholiki Georgiades in The Canadian Journal of Psychiatry

sj-docx-5-cpa-10.1177_07067437221114005 - Supplemental material for Provincial and Territorial Variation in Barriers in Accessing Healthcare for Children and Youth With Mental and Neurodevelopmental Health Concerns in Canada Click here for additional data file. Supplemental material, sj-docx-5-cpa-10.1177_07067437221114005 for Provincial and Territorial Variation in Barriers in Accessing Healthcare for Children and Youth With Mental and Neurodevelopmental Health Concerns in Canada by Jordan Edwards, Mahdis Kamali, Stelios Georgiades, Charlotte Waddell and Katholiki Georgiades in The Canadian Journal of Psychiatry
