# Supplementary material for: Provincial and Territorial Variation in Barriers in Accessing Healthcare for Children and Youth With Mental and Neurodevelopmental Health Concerns in Canada
Source: Can J Psychiatry. 2022 Aug 7;67(11):867–9. doi: 10.1177/07067437221114005 (PMC9561698; doi:10.1177/07067437221114005)
Supplement: sj-docx-1-cpa-10.1177_07067437221114005 - Supplemental material for Provincial and Territorial Variation in Barriers in Accessing Healthcare for Children and Youth With Mental and Neurodevelopmental Health Concerns in Canada [file sj-docx-1-cpa-10.1177_07067437221114005.docx]

***Appendix 1:*** Data Dictionary/Codebook.

| **Data Dictionary** | |
| --- | --- |
| ***Factor*** | **Coding** |
| **Demographics** | |
| ***Age*** | Age 1-11= 0 (Reference)  Age 12-17= 1 |
| ***Sex*** | Male= 0 (Reference)  Female= 1 |
| ***Family Composition*** | 2 biological parents in the home= 0 (Reference)  1 or no biological parents in the home= 1 |
| ***Income*** | Non-Low Income= 0 (Reference)  Low Income= 1  -Low-income indicator derived from Statistics Canada cut-offs of household income and size: <https://www150.statcan.gc.ca/t1/tbl1/en/tv.action?pid=1110023201> |
| ***Parental Education*** | High school or less= 0 (Reference)  More than high school, less than Bachelors= 1  Bachelors or more= 2 |
| ***Child Migrant Status***  ***(Child Foreign Born)*** | Non-migrant= 0 (Reference)  Migrant= 1 |
| ***Rurality*** | Rural= 0 (Reference)  Large Urban Centre= 1  Small Urban Centre= 2  -Based on Statistics Canada classifications. |
| ***Province*** | Classification of Territories was derived from people residing in the (Yukon, Northwest Territories, Nunavut). These were combined for vetting purposes. |
| **Clinical** | |
| ***MH Diagnosis*** | No Diagnosis= 0 (Reference)  Diagnosis= 1, Diagnosed by a health professional with at least one of the following conditions: Anxiety Disorder, Mood Disorder, or Eating Disorder |
| ***NDD Diagnosis*** | No Diagnosis= 0 (Reference)  Diagnosis= 1, Diagnosed by a health professional with at least one of the following conditions: Learning Disability/Disorder, ADD/ADHD, Autism Spectrum Disorder, Fetal Alcohol Spectrum Disorder |
| **Outcome: Barriers for Mental Disorders** | |
| ***Any Barriers for accessing health care for MH concerns*** | Did you experience difficulties accessing services for either/both of the following concerns (difficulties focusing or controlling behaviour, mental health issues)?  No MH Barriers = 0 (Reference)  Any MH Barriers= 1 |
| ***MH - Specific Barriers*** | What were the difficulties accessing services for either/both difficulties focusing or controlling behaviour, mental health issues? Multiple responses were possible for each respondent.   1. Wait time too long 2. Service not available in area 3. Cost 4. Told child not eligible 5. Other reason*   *The “other reason” category is a final catchall category in the barrier to access to care related questions. There was no free text option built into the survey. |
| **Outcome Barriers (Neuro-Development Disorders)** | |
| ***Any barriers for accessing health care for NDD concerns*** | Did you experience difficulties accessing services for either/both of the following concerns (speech or language difficulty, learning difficulties)?  No NDD Barriers= 0 (Reference)  Any NDD Barriers= 1 |
| ***NDD - Specific Barriers*** | What were the difficulties accessing services for either/both speech or language difficulty, learning difficulty? Multiple responses were possible for each respondent.   1. Wait time too long 2. Service not available in area 3. Cost 4. Told child not eligible 5. Other reason*   *The “other reason” category is a final catchall category in the barrier to access to care related questions. There was no free text option built into the survey. |
