# Supplementary material for: Provincial and Territorial Variation in Barriers in Accessing Healthcare for Children and Youth With Mental and Neurodevelopmental Health Concerns in Canada
Source: Can J Psychiatry. 2022 Aug 7;67(11):867–9. doi: 10.1177/07067437221114005 (PMC9561698; doi:10.1177/07067437221114005)
Supplement: sj-docx-2-cpa-10.1177_07067437221114005 - Supplemental material for Provincial and Territorial Variation in Barriers in Accessing Healthcare for Children and Youth With Mental and Neurodevelopmental Health Concerns in Canada [file sj-docx-2-cpa-10.1177_07067437221114005.docx]

***Appendix 2:*** Modified Poisson regression analyses exploring provincial and territorial variation in any/individual barriers to accessing care for mental health concerns.

| **Factors** | **Barriers to Access to Mental Health Care (Prevalence Ratios and 95%CIs), Sample denominator, children and youth requiring or receiving services for MH concerns n=4744*. *Estimated using weighted proportions.** | | | | | |
| --- | --- | --- | --- | --- | --- | --- |
|  | **Any Barriers** | **Wait times** | **Not Available** | **Cost** | **Eligibility** | **Other** |
| MH Diagnosis (ref none) | 11.47 (9.9, 13.28)* | 12.87 (10.73, 15.43)* | 12.74 (9.43, 17.21)* | 12.28 (9.55, 15.79)* | 16.97 (11.10, 25.94)* | 7.82 (5.76, 10.63)* |
| Age (ref <11) | 0.92 (0.79, 1.07) | 0.92 (0.76, 1.09) | 0.76 (0.57, 1.03) | 0.81 (0.62, 1.05) | 0.66 (0.41, 1.06) | 1.21 (0.89, 1.63) |
| Female sex (ref male) | 0.69 (0.60, 0.79)* | 0.65 (0.55, 0.78)* | 0.71 (0.54, 0.94)* | 0.79 (0.62, 1.00) | 0.48 (0.33, 0.71)* | 0.59 (0.46, 0.78)* |
| None or one biological parent in the home (ref two bio-parents) | 1.76 (1.54, 2.1)* | 1.75 (1.45, 2.12)* | 2.01 (1.46, 2.75)* | 1.81 (1.38, 2.37)* | 2.09 (1.29, 3.36)* | 2.36 (1.76, 3.16)* |
| Low Income (ref not low income) | 1.11 (0.94, 1.33) | 1.03 (0.84, 1.27) | 0.93 (0.64, 1.33) | 1.01 (0.74, 1.37) | 1.19 (0.69, 2.05) | 1.59 (1.17, 2.17)* |
| Parental education (ref high school or less)  1. between high school and bachelors,  2. bachelors + | 1.03 (0.86, 1.24)  1.09 (0.89, 1.33) | 0.98 (0.78, 1.24)  0.96 (0.75, 1.23) | 0.82 (0.56, 1.19)  0.94 (0.62, 1.43) | 1.02 (0.73, 1.43)  1.34 (0.94, 1.93) | 1.12 (0.64, 1.98)  1.33 (0.72, 2.48) | 1.73 (1.21, 2.47)*  1.95 (1.32, 2.88)* |
| Child Migrant Status (ref non-immigrant) | 0.30 (0.19, 0.47)* | 0.29 (0.16, 0.53)* | 0.26 (0.11, 0.64)* | 0.38 (0.19, 0.73)* | 0.28 (0.087, 0.89) | 0.25 (0.092, 0.69)* |
| Rurality (ref rural)  1.Large Urban Centre  2.Small Urban Centre | 0.96 (0.79, 1.16)  1.03 (0.85, 1.26) | 0.96 (0.75, 1.22)  0.99 (0.77, 1.29) | 0.43 (0.29, 0.62)*  0.87 (0.61, 1.22) | 1.21 (0.87, 1.68)  1.09 (0.76, 1.56) | 1.10 (0.64, 1.91)  1.08 (0.59, 1.97) | 1.16 (0.80, 1.67)  1.22 (0.83, 1.79) |
| **Contrast of predictive margins of provinces with 95%CIs** | | | | | | |
| Provinces (ref Canadian Mean)  Nfl  PEI  NS  NB Que  ON Man  Sas  Alb  BC  Territories | -0.0004 (-0.11, 0.10)  0.0058 (-0.005,0.017)  -0.0014 (-0.12, 0.0088)  0.0067 (-0.0048, 0.018)  0.0060 (-0.0014, 0.013  -0.0013 (-0.005, 0.0027)  -0.0034 (-0.014, 0.0068)  -0.0011 (-0.01, 0.008)  -0.0015 (-0.0086, 0.0055)  0.000059 ( -0.0067, 0.0068)  -0.0094 (-0.019, 0.00034) | 0.0030 (-0.0065, 0.13)  0.0097 (-0.001, 0.020)  0.00083 (-0.0081, 0.0097)  0.0086 (-0.0018, 0.19)  0.0072 (0.00060, 0.014)*  -0.00035 (-0.0038, 0.0031)  -0.0079 (-0.015,-0.00074)*  -0.0012 (-0.0089, 0.0066)  -0.0054 (-0.11, -0.00049)  -0.0021 (-0.0079, 0.0037)  -0.0123 (-0.019, -0.0055)* | 0.00011 (-0.0053, 0.0055)  0.0029 (-0.0026, 0.0084)  -0.000020 (-0.0054, 0.0054)  0.0013 (-0.0039, 0.0065)  -0.0037 (-0.067, -0.00070)*  0.000061 (-0.0021, 0.0022)  -0.00023 (-0.0063, 0.0059)  -0.0044 (-0.008, -0.00075)  -0.000011 (-0.0039, 0.0039)  0.0012 (-0.0029, 0.0053)  0.0029 (-0.0026, 0.0083) | -0.0071 (-0.011, -0.0035)*  0.0012 (-0.0058, 0.0082)  0.0030 (-0.0035, 0.0096)  -0.0011 (-0.0065, 0.0044)  -0.0019 (-0.0051, 0.0012)  0.0028 (0.00046, 0.0052)*  -0.00015 (-0.0061, 0.0058)  0.0025 (-0.0038, 0.0088)  -0.00047 (-0.0042, 0.0033)  0.0036 (-0.00078, 0.0079)  -0.0025 (-0.0077, 0.0027) | -0.0021 (-0.0049, 0.00067)  -0.00038 (-0.0040, 0.0033)  -0.00050 (-0.0044, 0.0034)  0.0013 (-0.0031, 0.0057)  0.00094 (-0.0019, 0.0039)  0.00085 (-0.00072, 0.00241)  -0.00084 (-0.0044, 0.0027)  -0.00086 (-0.0039, 0.0021)  0.00071 (-0.0019, 0.0034)  0.0035 (0.00031, 0.0067)*  -0.0026 (-0.0051, -0.00012)* | 0.0016 (-0.0050, 0.0081)  0.0012 (-0.0057, 0.0079)  0.00040 (-0.0056, 0.0064)  -0.00021 (-0.0062, 0.0058)  0.00058 (-0.0034, 0.0046)  -0.00064 (-0.0029, 0.0017)  0.0017 (-0.0052, 0.0086)  -0.0038 (-0.0089, 0.0014)  0.0036 (-0.0012, 0.0083)  0.00048 (-0.0036, 0.0046)  -0.0048 (-0.0091, -0.00048)* |
| All analyses were weighted and adjusted for self-reported diagnosis, age, sex, biological parents in the home, income, PMK education, migrant status of the child & rurality. | | | | | | |
