# Supplementary material for: Provincial and Territorial Variation in Barriers in Accessing Healthcare for Children and Youth With Mental and Neurodevelopmental Health Concerns in Canada
Source: Can J Psychiatry. 2022 Aug 7;67(11):867–9. doi: 10.1177/07067437221114005 (PMC9561698; doi:10.1177/07067437221114005)
Supplement: sj-docx-3-cpa-10.1177_07067437221114005 - Supplemental material for Provincial and Territorial Variation in Barriers in Accessing Healthcare for Children and Youth With Mental and Neurodevelopmental Health Concerns in Canada [file sj-docx-3-cpa-10.1177_07067437221114005.docx]

***Appendix 3:*** Modified Poisson regression analyses exploring provincial variation in any/individual barriers to accessing care for neuro-developmental concerns.

| **Factors** | **Barriers to Access to Neuro-developmental Care (Prevalence Ratios and 95%CIs) Sample denominator, children and youth requiring or receiving services for NDD concerns n=5577*. *Estimated using weighted proportions.** | | | | | |
| --- | --- | --- | --- | --- | --- | --- |
|  | **Any NDD Barriers** | **Wait times** | **Not Available** | **Cost** | **Eligibility** | **Other** |
| NDD Diagnosis (ref none) | 11.84 (10.21, 13.72)* | 10.89 (9.09, 13.06)* | 22.02 (15.45, 31.59)* | 15.95 (11.77, 21.61)* | 17.27 (12.06, 24.75)* | 14.90 (10.84, 20.49)* |
| Age (ref <11) | 0.44 (0.37, 0.51)* | 0.32 (0.26, 0.41)* | 0.48 (0.35, 0.65)* | 0.38 (0.28, 0.52)* | 0.53 (0.36, 0.79)* | 0.57 (0.42, 0.78)* |
| Female sex (ref male) | 0.77 (0.67, 0.89)* | 0.78 (0.65, 0.94)* | 0.79 (0.57, 1.09)* | 0.99 (0.89, 1.70) | 0.89 (0.61, 1.29) | 0.75 (0.54, 1.03) |
| None or one biological parent in the home (ref two bio-parents) | 1.02 (0.87, 1.19) | 1.00 (0.82, 1.23) | 1.25 (0.85, 1.84) | 1.24 (0.89, 1.71) | 1.04 (0.65, 1.65) | 0.93 (0.68, 1.27) |
| Low Income (ref not low income) | 1.18 (1.01, 1.38)* | 1.12 (0.92, 1.37) | 1.03 (0.68, 1.57) | 0.87 (0.65, 1.19) | 0.97 (0.62, 1.53) | 1.42 (1.03, 1.94)* |
| Parental education (ref high school or less)  1. between high school and bachelors,  2. bachelors + | 1.27 (0.98, 1.64)  1.21 (0.85, 1.73) | 1.13 (0.81, 1.57)  1.18 (0.75, 1.86) | 1.46 (0.73, 2.91)  1.03 (0.42, 2.49) | 1.57 (0.85, 2.92)  1.16 (0.53, 2.54) | 1.04 (0.48, 2.28)  0.67 (0.23, 1.94) | 1.50 (0.89, 2.55)  1.41 (0.68, 2.91) |
| Child Migrant Status (ref non-immigrant) | 0.55 (0.38, 0.79)* | 0.56 (0.35, 0.88)* | 0.94 (0.49. 1.80) | 0.83 (0.49, 1.39) | 0.13 (0.048, 0.33)* | 0.56 (0.24, 1.28) |
| Rurality (ref rural)  1.Large Urban Centre  2.Small Urban Centre | 0.98 (0.82, 1.17)  0.93 (0.77, 1.13) | 1.05 (0.84, 1.32)  0.93 (0.72, 1.19) | 0.42 (0.29, 0.62)*  0.77 (0.52, 1.13) | 1.68 (1.18, 2.39)*  1.08 (0.72, 1.63) | 0.97 (0.62, 1.54)  0.77 (0.46, 1.29) | 0.92 (0.62, 1.37)  1.05 (0.69, 1.57) |
| **Contrast of predictive margins of provinces with 95%CIs** | | | | | | |
| Provinces (ref Canadian Mean)  Nfl  PEI  NS  NB Que  ON Man  Sas  Alb  BC  Territories | -0.0004 (-0.11, 0.10)  0.0058 (-0.005,0.017)  -0.0014 (-0.12, 0.0088)  0.0067 (-0.0048, 0.018)  0.0060 (-0.0014, 0.013  -0.0013 (-0.005, 0.0027)  -0.0034 (-0.014, 0.0068)  -0.0011 (-0.01, 0.008)  -0.0015 (-0.0086, 0.0055)  0.000059 ( -0.0067, 0.0068)  -0.0094 (-0.019, 0.00034) | -0.00082 (-0.0096, 0.0079)  0.0091 (-0.00079, 0.19)  -0.0044 (-0.011, 0.0026)  0.0096 (-0.00011, 0.019)  0.0078 (0.0021, 0.0134)*  0.0016 (-0.0018, 0.0051)  -0.0060 (-0.014, 0.0016)  -0.0044 (-0.012, 0.0030)  -0.0081 (-0.013,-0.0033)*  0.0012 (-0.0050, 0.0074)  -0.0056 (-0.014, 0.0028) | 0.0015 (-0.0046, 0.0076)  0.0014 (-0.0036, 0.0065)  -0.0044 (-0.084,-0.00047)*  0.0037 (-0.0020, 0.0094)  -0.0049 (-0.076,-0.0023)*  -0.0021 (-0.042,-0.000011)*  0.00034 (-0.0064, 0.0071)  -0.0069 (-0.096,-0.0043)*  -0.0016 (-0.0053, 0.0019)  0.0021 (-0.0026, 0.0067)  0.011 (0.0023, 0.019)* | -0.0036 (-0.0093, 0.0020)  0.0098 (0.000032, 0.019)*  0.0042 (-0.0038, 0.012)  0.0012 (-0.0053, 0.0078)  -0.0010 (-0.0045, 0.0025)  0.00047 (-0.0020, 0.0029)  -0.0071 (-0.011,-0.0035)*  0.0026 (-0.0048, 0.010)  -0.0021 (-.0042, 0.0038)  -0.00021 (-0.0042, 0.0038)  -0.0043 (-0.0099, 0.0013) | 0.0054 (-0.00093, 0.012)  -0.00084 (-0.0047, 0.0030)  -0.00049 (-0.0049, 0.0039)  0.00074 (-0.0033, 0.0047)  -0.0012 (-0.0034, 0.0010)  -0.00018 (-0.0018, 0.0015)  -0.0021 (-0.0054, 0.0013)  -0.0013 (-0.0047, 0.0020)  0.0011 (-0.0019, 0.0042)  0.00073 (-0.0024, 0.0039)  -0.0019 (-0.0058, 0.0020) | -0.0029 (-0.0083, 0.0025)  -0.0059 (-0.0096,-0.0023)*  -0.0023 (-0.0071, 0.0025)  0.0010 (-0.0051, 0.0072)  -0.00072 (-0.0044, 0.0029)  -0.0023 (-0.0046,-0.000014)*  -0.00057 (-0.0073, 0.0062)  -0.0018 (-0.0074, 0.0039)  0.0028 (-0.0018, 0.0073)  0.0064 (0.0010, 0.012)*  0.0063 (-0.0023, 0.015) |
| All analyses were weighted and adjusted for self-reported diagnosis, age, sex, biological parents in the home, income, PMK education, migrant status of the child & rurality. | | | | | | |
