# Supplementary material for: Provincial and Territorial Variation in Barriers in Accessing Healthcare for Children and Youth With Mental and Neurodevelopmental Health Concerns in Canada
Source: Can J Psychiatry. 2022 Aug 7;67(11):867–9. doi: 10.1177/07067437221114005 (PMC9561698; doi:10.1177/07067437221114005)
Supplement: sj-docx-4-cpa-10.1177_07067437221114005 - Supplemental material for Provincial and Territorial Variation in Barriers in Accessing Healthcare for Children and Youth With Mental and Neurodevelopmental Health Concerns in Canada [file sj-docx-4-cpa-10.1177_07067437221114005.docx]

|  | **Prevalence of Diagnosis and Barriers to Access to Care for Mental Health Concerns, % (95%CI) (n=total sample) (wt%=weighted percentage)** | | | | | | | | | | | |
| --- | --- | --- | --- | --- | --- | --- | --- | --- | --- | --- | --- | --- |
|  | Qi: in the past 12 months did _ require or receive services for any of the following*:* “mental health issues”, “difficulties focusing or controlling behaviour” [mental health concerns] | | | | | | | | | | | |
| Variable | Canada  (n=47,871)  (wt%=100) | Newfoundland & Labrador (n=1,242)  (wt%=1.3) | Prince Edward Island  (n=1,441)  (wt%=0.4) | Nova Scotia  (n=1,438)  (wt%=2.3) | New Brunswick  (n=1,490)  (wt%=1.9) | Quebec  (n=3,977)  (wt%=22.2) | Ontario  (n=27,359)  (wt%=38.9) | Manitoba  (n=1,401)  (wt%=3.9) | Saskatchewan  (n=1,530)  (wt%=3.4) | Alberta  (n=3,505)  (wt%=13.4) | British Columbia  (n=3,504)  (wt%=12.0) | Territories  (n=984)  (wt%=0.5) |
| Diagnosed with a MH | 4.45  (4.26, 4.63) | 4.96  (3.75, 6.17) | 5.43  (4.26, 6.61) | 6.28  (5.03, 7.53) | 4.52  (3.46, 5.57) | 4.04  (3.43, 4.66) | 4.61  (4.37, 4.86) | 2.91  (2.03, 3.79) | 4.27  (3.25, 5.28) | 4.54  (3.85, 5.23) | 4.68  (3.98, 5.38) | 3.47  (2.32, 4.62) |
| Required or Received Services for MH | 9.91  (9.64, 10.17) | 10.36  (8.66, 12.05) | 10.65  (9.05, 12.24) | 13.50  (11.74, 15.27) | 12.47  (10.79, 14.15) | 11.45  (10.46, 12.44) | 8.82  (8.48, 9.15) | 6.63  (5.32, 7.93) | 9.34  (7.88, 10.80) | 10.25  (9.24, 11.25) | 10.29  (9.28, 11.29) | 8.81  (7.03, 10.59) |
| Any MH Barriers | 3.55  (3.38, 3.71) | 3.65  (2.60, 4.69) | 4.58  (3.49, 5.66) | 4.14  (3.11, 5.17) | 4.43  (3.38, 5.48) | 4.12  (3.50, 4.74) | 3.34  (3.13, 3.55) | 2.68  (1.83, 3.52) | 3.32  (2.42, 4.22) | 3.33  (2.74, 3.93) | 3.48  (2.87, 4.09) | 2.74  (1.72, 3.77) |
| Type MH Barriers  -Wait time too long    -Service not available  -Cost°  -Told child not eligible°  - Other reason | 2.41  (2.27, 2.55)  0.87  (0.78, 0.95)  1.06  (0.96, 1.15)  0.54  (0.48, 0.61)  1.06  (0.97, 1.15) | 2.85  (1.92, 3.78)  1.17  (0.57, 1.77)  0.93°  (0.67, 1.18)  0.49°  (0.30, 0.67)  1.20  (0.59, 1.81) | 3.65  (2.68, 4.62)  1.87  (1.17, 2.58)  0.93°  (0.67, 1.18)  0.49°  (0.30, 0.67)  1.16  (0.60, 1.71) | 3.07  (2.17, 3.96)  1.39  (0.78, 1.99)  0.93°  (0.67, 1.18)  0.49°  (0.30, 0.67)  1.31  (0.72, 1.89) | 3.43  (2.50, 4.35)  1.45  (0.84, 2.05)  0.93°  (0.67, 1.18)  0.49°  (0.30, 0.67)  1.18  (0.63, 1.73) | 3.05  (2.52, 3.59)  0.61  (0.37, 0.86)  0.75  (0.48, 1.02)  0.53°  (0.45, 0.62)  1.09  (0.77, 1.42) | 2.32  (2.14, 2.49)  0.87  (0.76, 0.98)  1.24  (1.10, 1.37)  0.53°  (0.45, 0.62)  0.98  (0.87, 1.09) | 1.34  (0.74, 1.95)  0.78  (0.32, 1.25)  0.79  (0.33, 1.26)  0.43°  (0.27, 0.59)  1.00  (0.48, 1.52) | 2.22  (1.48, 2.96)  0.54  (0.17, 0.91)  1.14  (0.61, 1.67)  0.43°  (0.27, 0.59)  0.62  (0.23, 1.02) | 1.84  (1.39, 2.29)  0.98  (0.65, 1.31)  0.92  (0.60, 1.24)  0.43°  (0.27, 0.59)  1.29  (0.92, 1.67) | 2.19  (1.71, 2.68)  1.02  (0.69. 1.36)  1.33  (0.95, 1.71)  0.78°  (0.52, 1.04)  1.06  (0.72, 1.40) | 1.28  (0.57, 1.98)  1.92  (1.06, 2.78)  0.63  (0.13, 1.13)  0.78°  (0.52, 1.04)  0.53  (0.07, 0.98) |
| °Indicate provinces were aggregated due to sample size limitations (i.e. provinces within a given regions were combined if any individual province contained cell sizes <5 for any of the individual types of barriers to access to care). Provincial aggregate groups: East (NS, NB, NFL, PEI), Central (ON, QUE), Prairie (SAS, MAN, ALB), West (BC, TERR). | | | | | | | | | | | | |

***Appendix 4:*** Descriptive statistics of the prevalence of diagnosis and barriers to accessing health care for mental health concerns.
