# Supplementary material for: Provincial and Territorial Variation in Barriers in Accessing Healthcare for Children and Youth With Mental and Neurodevelopmental Health Concerns in Canada
Source: Can J Psychiatry. 2022 Aug 7;67(11):867–9. doi: 10.1177/07067437221114005 (PMC9561698; doi:10.1177/07067437221114005)
Supplement: sj-docx-5-cpa-10.1177_07067437221114005 - Supplemental material for Provincial and Territorial Variation in Barriers in Accessing Healthcare for Children and Youth With Mental and Neurodevelopmental Health Concerns in Canada [file sj-docx-5-cpa-10.1177_07067437221114005.docx]

|  | **Prevalence of Diagnosis and Barriers to Access to Care for Neuro-Developmental Health Concerns, % (95%CI) (n=total sample) (wt%=weighted percentage)** | | | | | | | | | | | |
| --- | --- | --- | --- | --- | --- | --- | --- | --- | --- | --- | --- | --- |
|  | Qi: in the past 12 months did _ require or receive services for any of the following*:* “speech or language difficulties”, “learning difficulties”.[neuro-developmental health concerns] | | | | | | | | | | | |
| Variable | Canada  (n=47,871)  (wt%=100) | Newfoundland & Labrador (n=1,242)  (wt%=1.3) | Prince Edward Island  (n=1,441)  (wt%=0.4) | Nova Scotia  (n=1,438)  (wt%=2.3) | New Brunswick  (n=1,490)  (wt%=1.9) | Quebec  (n=3,977)  (wt%=22.2) | Ontario  (n=27,359)  (wt%=38.9) | Manitoba  (n=1,401)  (wt%=3.9) | Saskatchewan  (n=1,530)  (wt%=3.4) | Alberta  (n=3,505)  (wt%=13.4) | British Columbia  (n=3,504)  (wt%=12.0) | Territories  (n=984)  (wt%=0.5) |
| Diagnosed with a NDD | 11.14  (10.86, 11.43) | 11.22  (9.46, 12.98) | 12.61  (10.89, 14.32) | 13.54  (11.77,15.31) | 13.11  (11.39,14.82) | 15.81  (14.67,16.94) | 10.16  (9.79,10.51) | 6.54  (5.24, 7.83) | 8.35  (6.97,9.74) | 9.87  (8.88,10.86) | 8.69  (7.75, 9.62) | 7.91  (6.22, 9.61) |
| Required or Received Services for NDD | 11.65  (11.36, 11.94) | 12.79  (10.93, 14.66) | 12.39  (10.69, 14.10) | 15.68  (13.79,17.56) | 14.52  (12.73,16.32) | 12.56  (11.53,13.59) | 11.04  (10.67, 11.41) | 9.69  (8.14,11.25) | 11.21  (9.62,12.79) | 11.93  (10.86,13.01) | 10.97  (9.93,12.01) | 12.61  (10.52, 14.69) |
| Any NDD Barriers | 3.69  (3.52, 3.86) | 3.67  (2.62, 4.71) | 4.21  (3.17, 5.24) | 3.85  (2.86, 4.85) | 4.67  (3.60, 5.75) | 4.87  (4.19, 5.54) | 3.33  (3.12, 3.54) | 2.30  (1.52, 3.09) | 2.59  (1.79, 3.39) | 3.19  (2.60, 3.77) | 3.82  (3.19, 4.46) | 3.83  (2.63, 5.04) |
| Type NDD Barriers  -Wait time too long  -Service not available  -Cost  -Told child not eligible°  - Other reason | 2.39  (2.26, 2.53)  0.71  (0.64, 0.79)  1.04  (0.95, 1.13)  0.53  (0.47, 0.59)  1.02  (0.93, 1.11) | 2.14  (1.34, 2.95)  1.29  (0.66, 1.92)  0.64  (0.19, 1.08)  0.71°  (0.49, 0.92)  0.78  (0.29, 1.27) | 3.29  (2.37, 4.22)  1.77  (1.09, 2.45)  1.64  (0.98, 2.29)  0.71°  (0.49, 0.92)  0.53  (0.16, 0.91) | 2.01  (1.28, 2.73)  0.68  (0.26, 1.11)  1.48  (0.86, 2.11)  0.71°  (0.49, 0.92)  0.95  (0.45, 1.45) | 3.38  (2.46, 4.29)  1.92  (1.22, 2.61)  1.06  (0.54, 1.57)  0.71°  (0.49, 0.92)  1.30  (0.73, 1.88) | 3.65  (3.07, 4.23)  0.58  (0.34, 0.82)  1.21  (0.87, 1.55)  0.53°  (0.45, 0.61)  1.22  (0.87, 1.56) | 2.31  (2.13, 2.49)  0.65  (0.56, 0.75)  1.10  (0.98, 1.23)  0.53°  (0.45, 0.61)  0.76  (0.66, 0.86) | 1.30  (0.71, 1.89)  0.70  (0.26, 1.14)  0.27  (0, 0.55)  0.51°  (0.33, 0.68)  0.74  (0.29, 1.19) | 1.54  (0.92, 2.16)  0.22  (0, 0.45  1.03  (0.52, 1.54)  0.51°  (0.33, 0.68)  0.71  (0.29, 1.14) | 1.37  (0.98, 1.75)  0.72  (0.44, 0.99)  0.82  (0.52, 1.12)  0.51°  (0.33, 0.68)  1.24  (0.87, 1.60) | 2.04  (1.57, 2.51)  0.95  (0.62. 1.27)  0.96  (0.63, 1.28)  0.54°  (0.32, 0.75)  1.40  (1.01, 1.79) | 1.33  (0.61, 2.04)  2.12  (1.22, 3.03)  0.39  (0, 0.78)  0.54°  (0.32, 0.75)  1.36  (0.63, 2.08) |
| °Indicate provinces were aggregated due to sample size limitations (i.e. provinces within regions were combined if any individual province contained cell sizes <5 for any of the individual types of barriers to access to care). Provincial aggregate groups: East (NS, NB, NFL, PEI), Central (ON, QUE), Prairie (SAS, MAN, ALB), West (BC, TERR). | | | | | | | | | | | | |

***Appendix 5:*** Descriptive statistics of the prevalence of diagnosis and barriers to accessing health care for neuro-developmental health concerns.
